# The Influence of Gustatory and Olfactory Experiences on Responsiveness to Reward in the Honeybee

**DOI:** 10.1371/journal.pone.0013498

**Published:** 2010-10-20

**Authors:** Gabriela P. Ramírez, Andrés S. Martínez, Vanesa M. Fernández, Gonzalo Corti Bielsa, Walter M. Farina

**Affiliations:** Grupo de Estudio de Insectos Sociales, Departamento de Biodiversidad y Biología Experimental, Facultad de Ciencias Exactas y Naturales, IFIBYNE-CONICET, Universidad de Buenos Aires, Buenos Aires, Argentina; Royal Holloway University of London, United Kingdom

## Abstract

**Background:**

Honeybees (*Apis mellifera*) exhibit an extraordinarily tuned division of labor that depends on age polyethism. This adjustment is generally associated with the fact that individuals of different ages display different response thresholds to given stimuli, which determine specific behaviors. For instance, the sucrose-response threshold (SRT) which largely depends on genetic factors may also be affected by the nectar sugar content. However, it remains unknown whether SRTs in workers of different ages and tasks can differ depending on gustatory and olfactory experiences.

**Methodology:**

Groups of worker bees reared either in an artificial environment or else in a queen-right colony, were exposed to different reward conditions at different adult ages. Gustatory response scores (GRSs) and odor-memory retrieval were measured in bees that were previously exposed to changes in food characteristics.

**Principal Findings:**

Results show that the gustatory responses of pre-foraging-aged bees are affected by changes in sucrose solution concentration and also to the presence of an odor provided it is presented as scented sucrose solution. In contrast no differences in worker responses were observed when presented with odor only in the rearing environment. Fast modulation of GRSs was observed in older bees (12–16 days of age) which are commonly involved in food processing tasks within the hive, while slower modulation times were observed in younger bees (commonly nurse bees, 6–9 days of age). This suggests that older food-processing bees have a higher plasticity when responding to fluctuations in resource information than younger hive bees. Adjustments in the number of trophallaxis events were also found when scented food circulated inside the nest, and this was positively correlated with the differences in timing observed in gustatory responsiveness and memory retention for hive bees of different age classes.

**Conclusions:**

This work demonstrates the accessibility of chemosensory information in the honeybee colonies with respect to incoming nectar. The modulation of the sensory-response systems within the hive can have important effects on the dynamics of food transfer and information propagation.

## Introduction

A key organizational principle of most real-world networks such as metabolic and social networks is that the majority of members are involved in few interactions, while a very small number are responsible for most the connections [1]. Differences in connectivity strength among participants may occur within honeybee and other social insect colonies during foraging [2], [3]. When sharing the collected nectar, most nest mates participate in some oral contacts (trophallaxis events) to either receive or unload food [4], [5]. Thus, the average bee is involved in a few trophallaxis events, while a rather small number of workers participate in many trophallaxis events. This is the case for older pre-foraging worker hive bees that are approximately two weeks old and behave as food receivers. Their function is to unload freshly-collected food from incoming foragers and pass it on to other nest mates, an activity of crucial importance to ensure a fast flow of food and information within the hive [4], [6], [7]. In contrast with older pre-foraging bees, younger hive bees engage in fewer trophallaxis events [4], [8] probably because they are involved in other tasks (such as brood care and cell cleaning) located away from the area where nectar is brought into the hive [9]–[11].

Nectar flow inside the hive is greatly affected by bee prior experience [12]. In this sense, trophallaxis plays a central role in honeybee social cognition since it can modulate the response to rewards and associative learning in food-receiving individuals [13]–[16]. The information acquired during such social interactions could affect the decisions receivers make in different behavioral contexts [13], [17]. Thus, the incoming olfactory and gustatory information present in food might affect the response thresholds in a way that food-processing bees either accept or refuse to unload nectar from foragers, in turn strongly altering the nectar distribution and spread of information through the nest [5], [12], [15].

The coordination of the distribution of incoming nectar among hive mates may be explained by bees of different ages having a range of response thresholds for specific stimuli [18]–[20]. These differences in responsiveness may affect the display of particular behaviors. In order to understand how gustatory and olfactory cues associated with new resources (e.g. nectar or pollen sources) could affect this aspect of hive dynamics, the present study is focused on individual mechanisms related to response thresholds and olfactory memory retention.

By investigating sucrose response thresholds (SRTs) in honeybees, previous studies have established that the responsiveness to sugar could be a result of variation in genotype [21]–[23] and is strongly modulated by the sugar concentration of the circulating nectar [12], [16], [18], [19], [24], [25]. Under controlled conditions, workers in their first week of adult life, from colonies collecting highly concentrated sucrose solutions (50% sucrose) exhibit higher response thresholds to sucrose than those with access to lower concentration solutions (20% sucrose) [12]. In addition, nectar receiver bees (preforaging workers *ca.* two weeks old) also modify their SRTs according to the sucrose concentration received from the returning foragers, via trophallaxis [16]. However, within honeybee colonies a broad variability of gustatory responsiveness has been documented [12], [18], [25], [26]. These results suggest that the activities of foraging bees could modify the sucrose response thresholds of non-foraging bees while performing different tasks throughout the colony.

This is of particular importance when it is considered that the initial high proboscis extension response (PER) after a single pairing between an odor (conditioned stimulus, CS) and a sugar solution reward (unconditioned stimulus, US) is dominated by a sensitization component [27], leading to a transient increase in the PER to many stimuli. Therefore, it is expected that this non-associative component can influence the gustatory responsiveness of the conditioned bees differently according to the age and/or the task being performed. As gustatory response score (GRS) is inversely related to SRT, this behavior can be quantified by the SRT procedure [21], [23], which consists of quantifying the GRS of each bee, after presenting them with sucrose solutions of increasing concentration (see methods for details). The possibility that not all age classes of hive bees respond in the same way to changes in food chemosensory information may be related to different sensitivity levels towards the stimuli. The variability of sensory-response systems in bees involved in foraging-related tasks can affect how the division of labor is regulated among hive bees. In this context, we investigated whether hive bees of different ages modify their SRTs, following sudden changes in gustatory and olfactory information from the food they encounter. Additionally, olfactory memories established during the distribution of scented food throughout the hive were evaluated using the PER paradigm to test bees that had already established specific associations between an odor and sucrose solution transferred through mouth-to-mouth trophallactic contact [13], [15]. By using this procedure, we were able to determine how scented nectars affect the olfactory responses of bees involved in the distribution of hive nectar.

To evaluate the gustatory responsiveness and memory retention we used worker bees of two age classes: one-and two-week-old bees corresponding to bees involved in nursing or food-processing tasks respectively [9]–[11]. Bees of these age classes, reared either in laboratory or in in-hive conditions were evaluated under controlled conditions after having experienced different reward programs (an increase or a decrease in the sucrose content of the food offered) and olfactory stimuli (odors either diluted in their food or volatile odors present only in the air of their rearing environment). Finally, to determine the acceptance threshold of the scented sucrose solutions within the colony, we also studied the trophallaxis events among nest mates once the scented food started circulating and subsequently after it was withdrawn.

The present experimental series allowed us to test three different hypotheses: (1) GRSs of pre-foragers will be modified once a change in gustatory information of the handled food occurs. This modulation would depend on the age of pre-forager honeybees; (2) GRSs will be modified by the odor cues associated with circulating nectar but not the rearing environment and (3) the number of social interactions among nest mates will be affected by the presence of scent in the circulating food. To obtain a general picture of the sensory-response system of bees throughout the nest while the food circulates, we simultaneously analyzed responsiveness to sucrose and olfactory memory retrieval of hive bees belonging to different age classes at different feeding times.

## Materials and Methods

### Study site and animals

The experiments were performed between January and June 2005, 2006 and 2007, during the austral summer-fall season, at the experimental field of the University of Buenos Aires, Argentina (34° 32′ S, 58° 26′ W).

Worker bees where either reared in the laboratory under controlled conditions or captured at an observational colony. The workers reared under controlled conditions were obtained from sealed brood frames placed in an incubator (36° C, 55% relative humidity (RH) and darkness). After emerging, young workers (0–1 days old) were collected in groups of about 120 individuals in wooden cages (10×10×10 cm) with a wire mesh door on one side. A 15% weight/weight (w/w) sucrose solution was offered in the cages in addition to water and pollen *ad libitum*. Caged bees were kept in an incubator (30° C, 55% RH and darkness) until the testing day. Bees subjected to the odor exposure experiment were placed in a third incubator during the last 24 h prior to testing (see below for details).

In addition, workers were also obtained from an observational colony (reduced to a two-frame hive containing a mated queen, brood and about 4000 workers). At the beginning of the experiment, recently emerged bees (0–1 days old) obtained from sealed brood in the incubator (36° C, 55% relative humidity and darkness) were marked with different colors of acrylic paint (ALBA-Argentina), to determine their age at a later stage. Bees were then introduced into the observational hive and were readily accepted by the rest of the colony [29]. Forager bees were captured while feeding on a plate (∼8 cm in diameter) offering a sucrose solution (15% w/w) a few centimeters from the entrance of the hive. Bees ranging from 4–9 and 12–16 days old were captured from the experimental hive by means of a Perspex device with sliding walls (for details see [15]). One side of the device slid horizontally, allowing us to scan the area of the exposed face of the hive, while a second side (4×27 cm) inserted into the previous one could be moved vertically. The vertically sliding door contained a plastic tube (3.5 cm diameter) to allow the insertion of a suction tube to capture a marked bee. During the entire experimental period the colony was placed inside a flight chamber (6×3×2 m) that remained open between the experiments allowing bees to fly freely, but was kept closed while testing behavioral responses.

### Gustatory responsiveness (PER-GRS assay)

In order to measure gustatory responsiveness, the antennae of test bees were stimulated with droplets of sucrose solution of increasing concentration [21]. The captured bees were anesthetized with CO_2_ and mounted in small metal tubes that restrained body movement but allowed free movement of antennae and mouthparts [30]. After awakening, bees were offered water to drink and housed in an incubator (30°C, 55% RH and darkness) for at least 1.5 h before assessing their response. Prior to the PER-GRS assay, water was offered again in order to avoid confounding thirst effects. Bees were assayed by presenting sucrose solutions of increasing concentration (0.1, 0.3, 1, 3, 10, 30 and 50% w/w). The lowest sucrose concentration at which an individual responded by extending its proboscis was interpreted as its SRT. Bees were lined up in groups of 20–30 individuals and tested for each concentration sequentially: i.e. all bees were tested first at 0.1%, then at 0.3%, and so on [21]. Between each concentration of sucrose solution, all bees were tested for their response to water. This controlled for potential effects of repeated sucrose stimulation that could lead to increased sensitization or habituation. The inter-stimulus interval between water and sucrose solution varied between 2 and 4 min depending on the number of individuals tested at a given time. At the end of the procedure, a gustatory response score (GRS) was obtained for each bee. This score was based on the number of sucrose concentrations to which the bees responded (correlating with the SRT since bee normally responds to all concentrations above their threshold [12], [23]). The response was arbitrarily quantified with scores from one to seven, where one represented a bee that only responded to one concentration of sucrose (usually the 50% w/w), while a score of seven represented an individual that responded to all concentrations tested. If a bee failed to respond to a sucrose concentration in the middle of a response series (e.g. responded to 0.1, 0.3, 3 and 10%, but did not respond to 1%), this ‘failed’ response was considered to be an error and the bee was deemed to have responded to that concentration as well. A bee that did not respond to any of the sucrose concentrations (score of 0) was excluded from further analyses [16], [31]. In addition, those bees that responded to all sucrose concentrations and all presentations of water were excluded from analyses as they appeared not to be able to discriminate between sucrose solution and water.

### Olfactory memory test

After a bee associates an odor with a reward, the presentation of this odor alone triggers the proboscis extension response, i.e. the conditioned response (CR) [26], [28]. To measure this response, a syringe containing either a clean filter paper (control: 30×3 mm) or a filter paper soaked with 4 µl of pure odor (Linalool, LIO or Phenylacetaldehyde, PHE, Sigma-Aldrich) was connected to an air pump creating a flow of 2.5 ml/s. The airflow was directed to the antennae of a harnessed bee. Only bees that showed an unconditioned response (extended their proboscis after applying 50% w/w sucrose solution to the antennae) and did not respond to the mechanical air flow stimulus were tested. Stimulus presentation lasted for 46 s and consisted of 20 s of clean air, 6 s of odor, and 20 s of clean air again. The volatile compounds used (LIO and PHE) are natural components of flower odors [32].

### Experimental procedure

#### Response to varying sucrose concentration

The aim of this experiment was to evaluate the effect of changes in sucrose concentration on the gustatory responsiveness of young and old pre-foraging bees. PER-GRS assays were performed on 7- (young) and 14- (old)-day-old bees. Caged bees were exposed to a constant reward program of unscented sucrose solution (15% w/w) after eclosion, except during the last 24 h (i.e. on the seventh or fourteenth day depending of the age group). During these last 24 h, caged bees were fed with either a diluted sucrose solution (3% w/w, decreasing reward program) or a concentrated sucrose solution (30% w/w, increasing reward program). Both sucrose concentrations were offered also during the PER-GRS assay (see above). To avoid offering a diet too poor in sugar throughout the whole experimental period, we fed bees with 15% w/w instead the intermediate sucrose solution (10% w/w) determined by the concentrations tests in the PER-GRS assays. Experimental bees were removed from the cages and their GRSs were measured at different times with respect to replacing the 15% w/w sucrose solution with either diluted or concentrated solutions (immediately before replacing the solution, 0 h; six hours after the replacement and 24 h later).

#### Response to scented food

The aim of this experiment was to test for effects of volatile stimuli on the gustatory responsiveness of young and old pre-foraging bees. PER-GRS and odor memory assays were conducted on 7- and 14-day-old bees reared in cages and exposed to odor for 24 h. While the sucrose concentration was kept constant during the experimental period (15% w/w), during the last 24 h in the cages, bees were presented with: (i) an unscented sucrose solution, (ii) a scented sucrose solution (50 µl LIO/L solution; it is assumed that feeding from a scented sucrose solution leads to associative learning in honeybees, see [13], [15], [33]), or (iii) an unscented sucrose solution with LIO presented as a volatile in the rearing environment (via a filter paper with 150 µl of LIO, [34]). To reduce odor accumulation in cages, air was replaced regularly with a pump.

Experimental bees were removed from the cages and their GRS and conditioned responses (CRs) measured in the PER setup, before the presentation of the new solutions (0 h), at six hours and 24 h after changing the odor conditions (stimulation times). The ingested volume of sucrose solution was also recorded for each stimulation time and for each age class. The sucrose solutions were delivered to bees through a volumetric test tube, so the total ingested solution could be quantified. The total volume of sucrose solution ingested was divided by the number of bees housed in the group.

#### Response to food scent withdrawal

The aim of this experiment was to assess the duration of the “food-scent effect” on bee responsiveness to the reward and memory retention. GRSs and CRs of two-week-old bees were measured in the PER setup after removal of the scented sucrose solution. An unscented solution (15% w/w) was made available during the first 13 days, and then replaced by a scented one (50 µl LIO/L solution) for 24 h, after which the unscented solution was offered again. The behavioral response was tested at zero hour and 24 h after introduction of the scented sucrose solution, and subsequently six and 24 h after withdrawing the scented food source (i.e. 30 and 48 h after the introduction of the scented solution, respectively). The same procedure was repeated for another group of caged bees, but in this case the unscented sucrose solution (15% w/w) was presented throughout the whole experimental period (i.e. 15 days).

#### Response of free-flying bees to scented/unscented food

The effect of scented food on gustatory responsiveness was also studied under natural conditions in an experimental colony. Foraging bees from the observation hive were trained to an artificial feeder offering either an unscented sucrose solution (15% w/w) or a LIO-scented sucrose solution (50 µl LIO/L of 15% w/w sucrose solution) for 8 and 24 hours. During these periods, bees were not allowed to exit the flight chamber. Hive bees of different ages (6–9 days old, and 12–16 days old) and bees at the artificial feeder (henceforth: foragers; the minimum age at which workers initiate foraging is approx. 17–20 days, [9]–[11]) were captured. This procedure was repeated for alternating 8 h- and 24 h-periods for unscented and scented sucrose solutions. To minimize the effects of prior olfactory experiences on gustatory responsiveness and memory retention, the colony was exposed to a solution-odor sequence: the unscented solution was presented for 8 h on day 1, the scented solution was presented for 8 h on day 2. This procedure was repeated twice, and the minimal interval between scented solution periods was five days. Ten days from the beginning of the experiment, the unscented solution was presented again for 24 h, and the LIO-scented sucrose solution was presented for 24 h the following day. In one group of bees, the GRSs and CRs were tested in the PER setup after unscented food was offered to the colony (i.e. 0 h of stimulation time) and after eight hours or 24 h of scented food flow, the same measurements were taken from a second group of workers.

We scan sampled the number of trophallaxis events within the hive when unscented or scented sucrose solution was being passed on between nest mates in the colony. Response variables were recorded within a rectangular area (4cm×8cm) of the frame (selected at random) during a 90-s period. Sampling was repeated 8 times within a two-hour period after the introduction of the scented or unscented sucrose solutions. The trophallactic activity was represented as the number of trophallaxis events per bee during a 10 min-period and was measured at the same time as either unscented or scented food flowed into the colony.

### Statistical analysis

GRSs data were treated as nonparametric because the assumption of homogeneity of variance was not met. Median GRSs were compared between stimulation times within each age class and under a specific reward program [35] using Kruskal-Wallis (K-W) ANOVA tests followed by Dunn comparisons between groups. Mann-Whitney *U* tests [36] were used to compare GRSs between individuals that had responded positively or negatively in the PER paradigm to food odor, within each age group. G-tests were used to compare percentages of bees showing PER in response to scented sucrose solution or to a novel odor between the sub-groups in both experimental conditions. When significant differences were found, multiple planned comparisons between each group were performed with Dunn Sidak significant level correction [α' = 1−(1−α) ^1/k^, k = number of comparisons] [35]. Volumes of ingested sucrose solution were compared between reward programs in each age class with repeated measures ANOVA. This parametric analysis was conducted as the assumption of homogeneity of variance was satisfied. Trophallaxis behavior was compared between groups within each stimulation time using a Mann-Whitney *U* test [36].

## Results

### Gustatory responsiveness after changes in sucrose concentration

**Figure 1:**
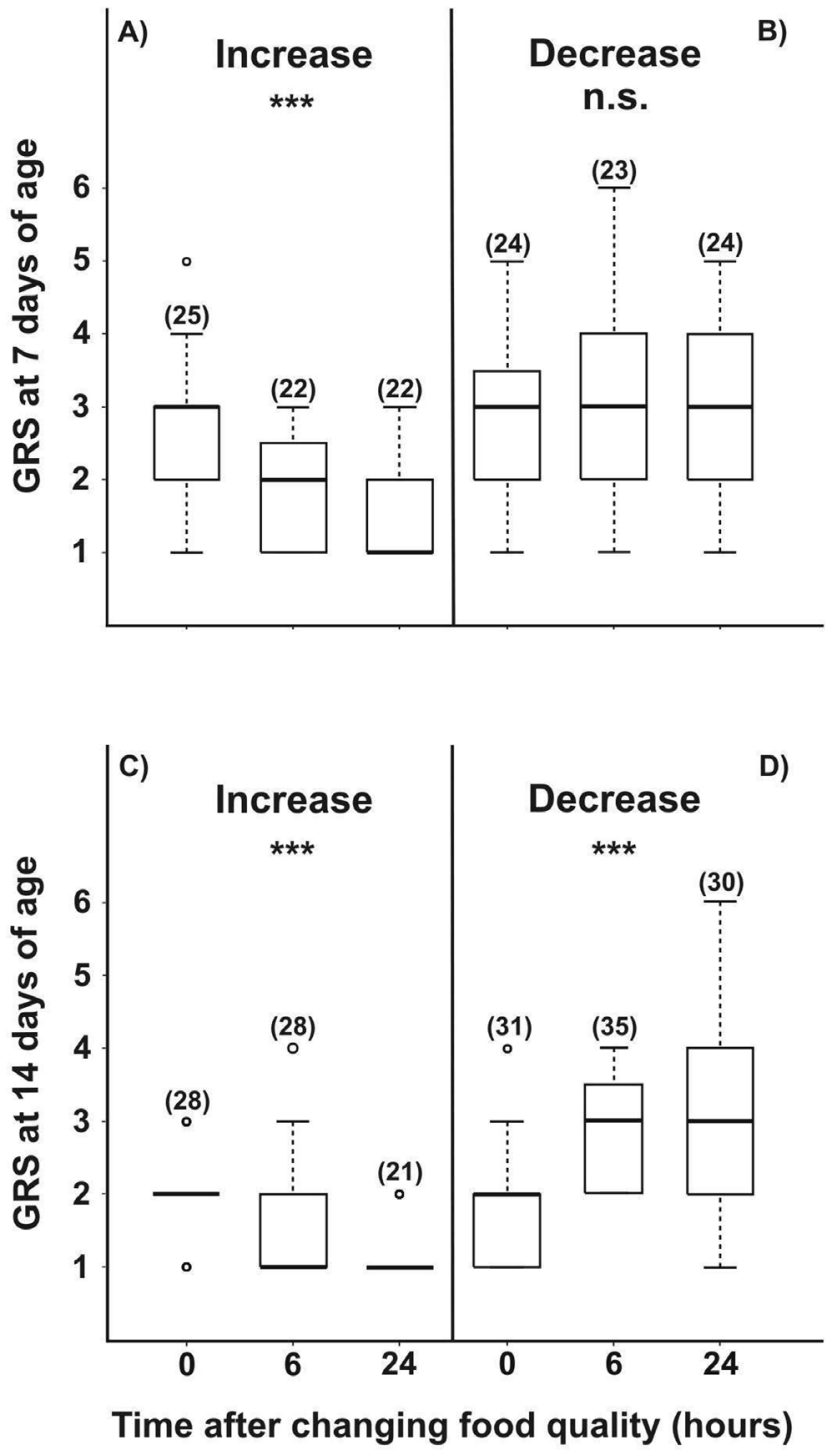
Gustatory responsiveness after changes in sucrose concentration. Prior to tests, caged adult bees were fed a constant reward program of unscented 15% w/w sucrose solution and their gustatory response scores (GRSs) were measured. At 7 days old, bees were fed either with an increased (**A**, 30% w/w, *increasing reward program*) or a decreased (**B**, 3% w/w, *decreasing reward* program) concentration of sucrose solution. Similar reward programs were offered to 14-day-old bees (**C** and **D**). Zero, 6 and 24 h columns in each panel represent the time period which elapsed between the change in sucrose concentration and testing. The number of bees tested is shown in parentheses. Boxes indicate the inter-quartile range, horizontal lines within boxes indicate the medians, whiskers include all points within 1.5 times the inter-quartiles, empty circles indicate outliers. (Kruskal-Wallis, ***P<0.0001, n.s. not significant).

Seven day-old bees reared under laboratory conditions decreased their GRS after an increase in sucrose concentration, while no changes over time were found after a decrease in concentration (increasing reward program: K–W: H = 20.61, p<0.001, N = 69; decreasing reward program: K–W: H = 0.8, p = 0.67, N = 71, Fig. 1A and B). In contrast, the GRS of 14-day-old bees changed in response to both an increase and decrease in sucrose content. 14-day old bees decreased their GRSs after an increase in the sucrose concentration of the food (K–W: H = 19.55, p<0.001, N = 77, Fig. 1C), and increased their GRSs after a decrease in sucrose content (K–W: H = 20.39, p<0.001, N = 96; Fig. 1D). Although responses to sucrose in young bees varied according to changes in the reward programs, these responses were different for the two age classes studied.

### Gustatory responsiveness to scented food

**Figure 2:**
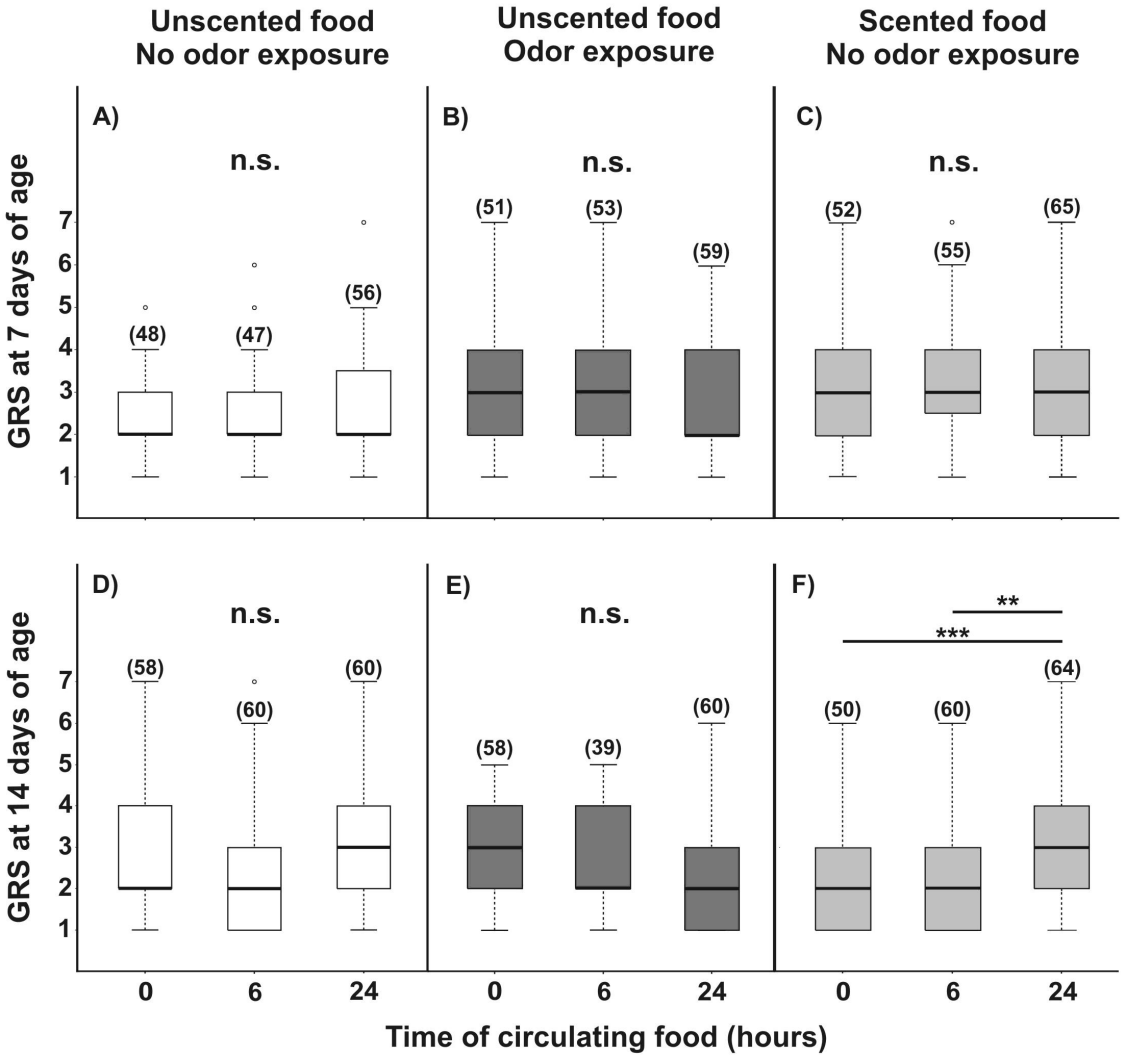
Gustatory responsiveness after changes in odor. Gustatory response scores (GRSs) of caged bees fed on 15% w/w sucrose solution throughout their adult life. Groups of 7- and 14-day-old bees experienced one of three treatments: i) fed unscented 15% w/w sucrose solution (**A** and **D**, unscented food - no odor exposure: open boxes), ii) fed unscented 15% w/w sucrose solution with linalool (LIO) as a volatile in the rearing environment (**B** and **E**, unscented food - odor exposure: dark gray boxes), or iii) fed LIO scented 15% w/w sucrose solution (**C** and **F**, scented food - no odor exposure: light gray boxes). Zero, 6 and 24 h columns in each panel represent the time for which the treatment specific food type was circulating in the hive prior to GRS testing. Asterisks indicate statistical differences (Kruskal-Wallis, ** p<0.01, n.s. not significant; for details see the text). Boxes indicate the inter-quartile range, horizontal lines within boxes indicate the medians, whiskers include all points within 1.5 times the inter-quartiles, empty circles indicate outliers. The number of observations is shown in parentheses.

Seven-day-old bees exposed to no odor in the rearing environment (laboratory conditions), did not differ in their GRSs throughout stimulation time when unscented food was offered to them (K–W: H = 2.38, p = 0.304, N = 151, Fig. 2A). Similarly, when bees of this age class were exposed to odor (LIO) in the rearing environment, the GRSs were not affected during the exposure period (K–W: H = 5.41, p = 0.067, N = 163; Fig. 2B). Likewise, the GRSs did not differ among stimulation times in bees exposed to no odor in the rearing environment but fed with scented sucrose solution (K–W: H = 5.51, p = 0.064, N = 172; Fig. 2C). The GRSs of 14-day old (older pre-foraging) bees did not appear to be affected when fed with an unscented sucrose solution with (K–W: H = 2.91, p = 0.23, N = 178; Fig. 2D) or without LIO odor in their rearing environment (K–W: H = 4.79, p = 0.091, N = 157; Fig. 2E). In contrast, bees of this age class showed an increase in the GRS after 24 h of stimulation with scented food (K–W: H = 10.06, p = 0.0065, N = 174; Dunn contrast 0 h vs. 24 h: Q = 3.57, p<0.001, N = 114; Dunn contrast 6 h vs. 24 h: Q = 2.82, p<0.01, N = 124; Fig. 2F).

**Figure 3:**
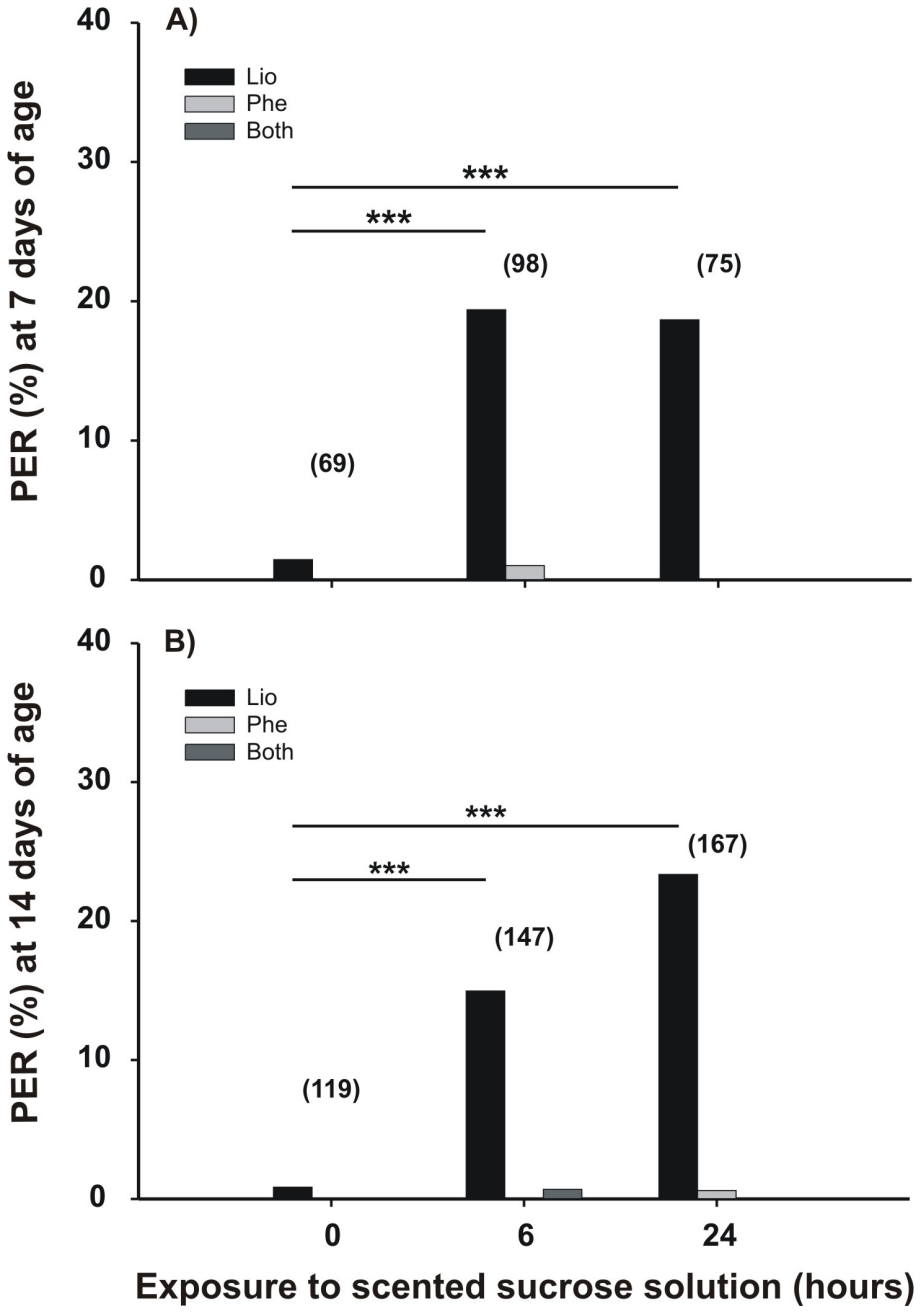
Memory retention after exposure to scented food. Prior to tests, caged adult bees were fed a constant reward program of unscented 15% w/w sucrose solution. Either at 7 days old (**A**) or 14 days old (**B**), percentage of bees that extended their proboscis upon the first presentation of an odor (% PER) was calculated for: the solution odor (linalool, LIO, gray bars), a novel test odor (phenylacetaldehyde, PHE, dark gray bars), or both (white bars) in bees that were fed for 24 hours with a scented 15% w/w sucrose solution. Asterisks indicate statistical differences (G-test for, ** p<0.01; for details see the text). The number of observations is shown in parentheses.

We also evaluated the PER after different durations of exposure to the odor (a measurement of the CR). Results showed that 7-day-old bees increased their PER frequencies to the solution odor (LIO) throughout the stimulation period (G test: G = 17.39, p = 0.0002, N = 242, Fig. 3A). Multiple comparisons allowed us to detect statistically significant differences between 0 h and 6 h after scenting the solution with LIO (p<0.0001; Fig. 3A) and between 0 h and 24 h (p<0.0001; Fig. 3A), but not between 6 h and 24 h (p>0.5; Fig. 3A). PER frequencies to LIO (solution odor) increased in 14-day-old bees (G test for: G = 38.49, p<0.0001, N = 433; Fig. 3B), while we observed minimal PER levels in tests with the novel odor (PHE) and a mixture of both odors (LIO and PHE). Multiple comparisons showed statistical differences between 0 h and 6 h after scenting the solution with LIO (p<0.0001; Fig. 3B) and between 0 h and 24 h (p<0.0001, Fig. 3B), but not between 6 h and 24 h (p>0.05, Fig. 3B).

Since differences in the GRS were detected depending on the age at which the caged bees were tested, we compared the GRSs for 7 and 14 day old bees at the point when LIO-scented sucrose was added (i.e. all 0 h scores were defined as an unscented situation). The GRS of 7 day old bees was significantly higher than for 14 day old bees (7-day-old bees, GRS median = 3, N = 151 and 14-day-old bees, GRS median = 2, N = 166, Mann-Whitney U test, U = 4320.5, p = 0.013).

In addition, for the LIO-scented sucrose solution treatment we compared the GRS of 7 day old bees which showed a positive response (extended their proboscis) to the odor, with those bees which showed no response (i.e., responses of bees tested after 6 h and 24 h were pooled). The same comparison was made for 14 day old bees. No statistical differences between groups were found for 7-day-old bees (Mann-Whitney test, U = 1413.5, p = 0.0992, N = 172). In contrast, 14-day-old bees had higher GRSs when considering those that showed the PER towards the odor (LIO) in the solution (Mann-Whitney test, U = 1610, p<0.0005, N = 185).

It is worth mentioning that bees of both age classes did not differ significantly in the amount of sucrose solution they ingested when feeding from the unscented or scented solutions (for 7-day-old bees: F_1,5_ = 0.69, p = 0.44, N = 7; for 14-day-old bees: F_1,13_ = 0.62, p = 0.46, N = 15, two-way ANOVA for repeated measures), suggesting similar levels of satiation for bees in both treatments.

### Gustatory responsiveness to food scent withdrawal

**Figure 4:**
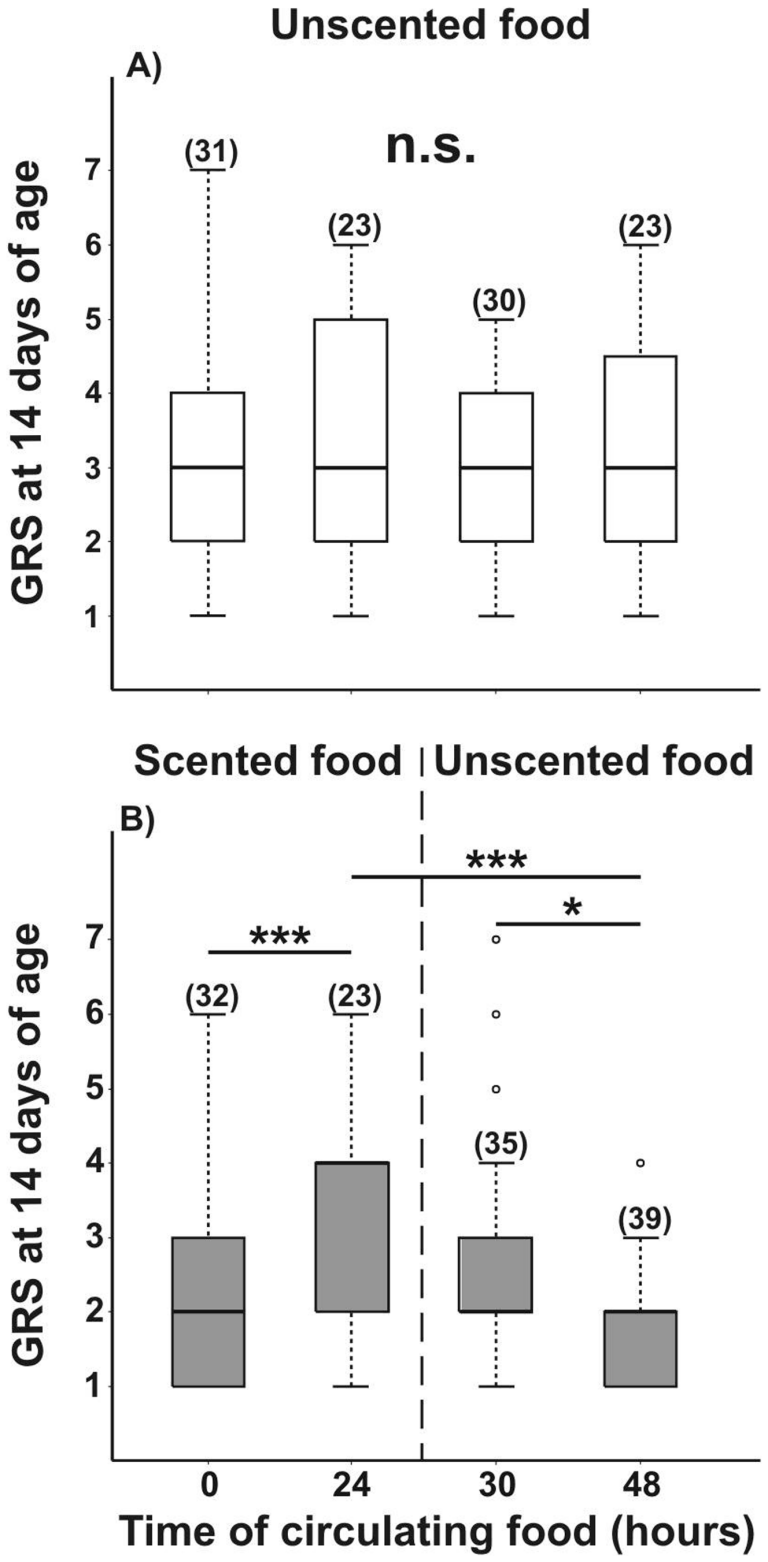
Gustatory responsiveness after withdrawl of scented sucrose solution. Gustatory response score (GRS) of caged bees that were exposed during their whole adult lifespan (15 days) to a constant reward program of unscented 15% w/w sucrose solution (**A**). (**B**) GRSs of bees that were fed until 14 days of age with an unscented solution, then fed scented sucrose (LIO, 15% w/w) for 24 hours, and then again on unscented sucrose for the following 24 hours (i.e. 15th day). Asterisks indicate statistical differences (Kruskal-Wallis test, * p< 0.05, ** p<0.01, n.s. not significant; for details see the text). The number of observations is shown in parentheses. Boxes indicate the inter-quartile range, horizontal lines within boxes indicate the medians, whiskers include all points within 1.5 times the inter-quartiles, empty circles indicate outliers.

**Figure 5:**
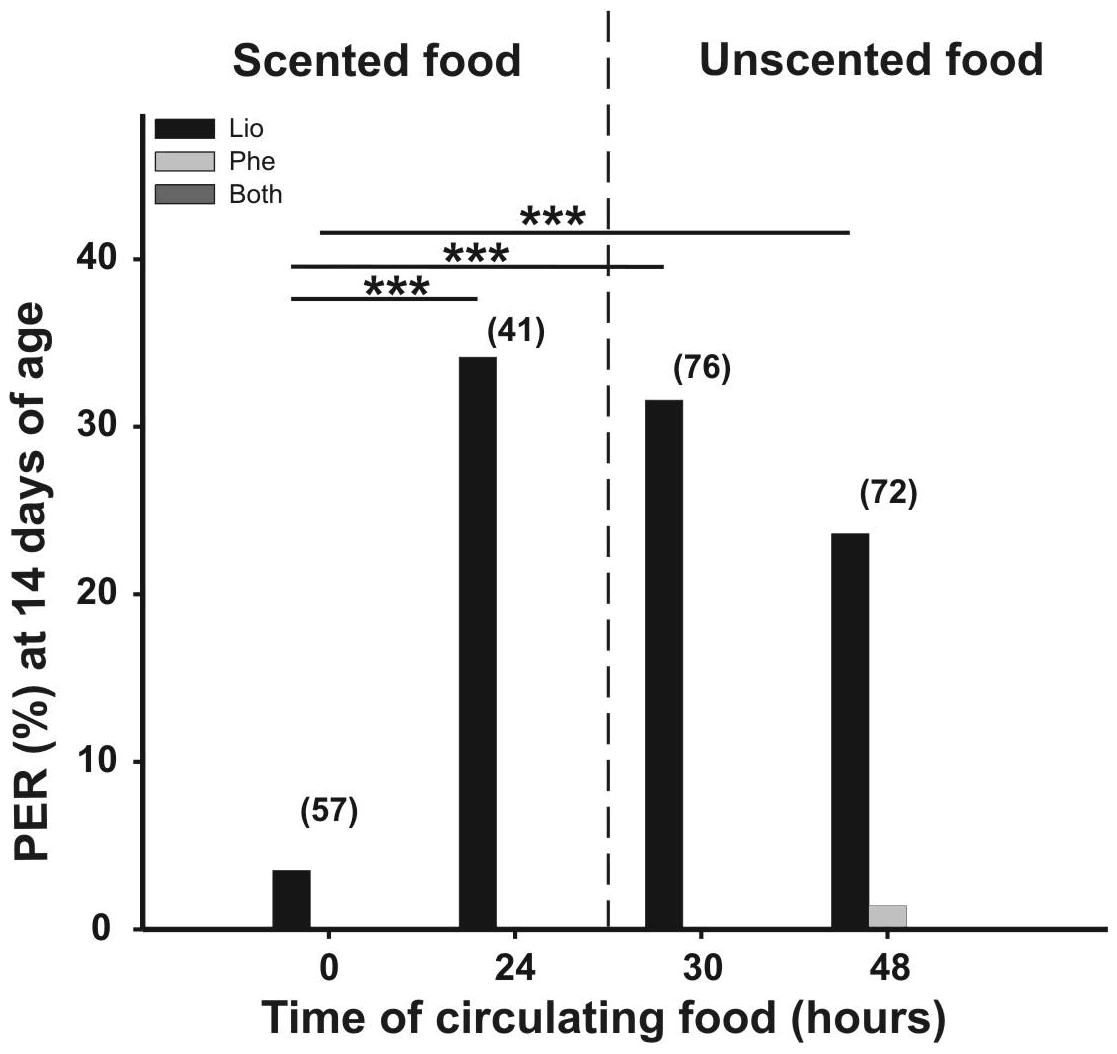
Memory retention after withdrawl of scented sucrose solution. Bees were fed until 14 days of age with an unscented solution, then fed for 24 h with a scented sucrose solution (LIO, 15% w/w)and on the 15th day once again fed with the unscented solution. The figure shows the percentage of bees that extended their proboscis on the first presentation of the odor for the solution odor (linalool, LIO, black bars), a novel test odor (phenylacetaldehyde, PHE, gray bars), or both (dark gray bars). The asterisks indicate statistical differences in a G-test (** p<0.01, for details see the text). The number of observation is shown in parentheses.

Since only the 14-day-old caged honeybees showed an increase in their GRSs after being exposed to LIO-scented food for 24 h, the duration of this increased response was further investigated in bees of this age. GRSs of 14 day-old bees fed with an unscented sucrose solution did not differ over the 48 hour timescale tested (K–W: H = 0.514, p = 0.916, N = 107, Fig. 4A). In contrast, bees exposed to LIO-scented food showed evidence of changes in GRS (K–W: H = 13.24, p = 0.004, N = 131 Fig. 4B). Indeed, the GRSs of these bees increased significantly during 24 hours of stimulation (Dunn comparison: p<0.001; Fig. 4B left), a result consistent with those shown in Fig. 2F. However, these values significantly decreased 48 h from the beginning of stimulation, i.e. 24 h after withdrawing the LIO-scented food (24 h vs. 48 h: Dunn comparison: p<0.001; Fig. 4B right). Moreover, these GRSs were lower than GRSs of bees tested six hours after withdrawing the LIO-scented food (i.e., 30 h from the beginning of stimulation; Dunn comparison: p<0.05; Fig. 4B right). No significant differences were found between scores after 24 h of stimulation with LIO-scented food versus scores of bees tested six hours after replacing the scented solution by an unscented one, i.e. after 30 hours (Dunn comparison: p>0.2; Fig. 4B). This indicates that six hours after removal of the LIO-scented sucrose solution, GRSs had not yet dropped significantly from the level observed after 24 hours of exposure. However, GRSs did subsequently decrease between 6–24 hours after the LIO-scented sucrose was replaced by an unscented solution. In contrast, the elevated PER levels to the solution odor recorded after 24 hours of exposure to LIO-scented sucrose solution had not decreased significantly from this level 24 hours after withdrawing this scent cue (G-test: G = 22.86, p<0.00001, N = 246, Dunn comparison: 0 h vs. 24 h p<0.0001, 0 h vs. 30 h p<0.0001 and 0 h vs. 48 h p<0.001; Fig. 5).

### Gustatory responsiveness of free-flying bees to scented and unscented food

**Figure 6:**
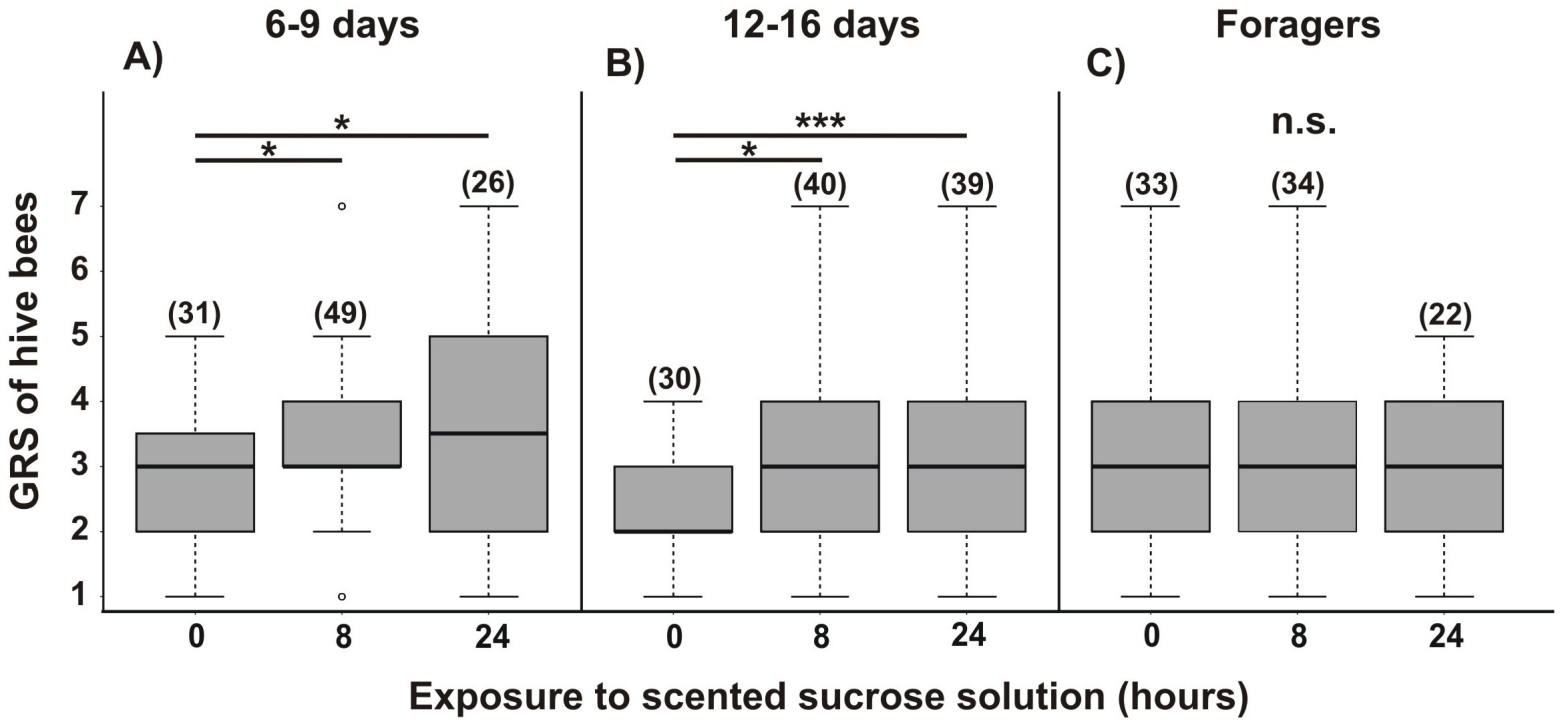
Gustatory responsiveness of free-flying bees after exposure to scented sucrose solution. GRSs of hive bees belonging to different age groups: 6/9 days old (**A**), 12/16 days old (**B**) and foragers (**C**) were measured. Bees were captured either while being offered an unscented sucrose solution (15% w/w) (0 hours) or after 8 h and 24 h of foraging from a scented sucrose solution (LIO, 15% w/w). The asterisks indicate statistical differences in a Kruskal-Wallis test and Dunn comparison (* p<0.05, ** p<0.001, n.s. not significant; for details see the text). The number of observations is shown in parentheses. Boxes indicate the inter-quartile range, horizontal lines within boxes indicate the medians, whiskers include all points within 1.5 times the inter-quartiles, empty circles indicate outliers.

Within the social context of the hive, the effect of odor cues was investigated in worker bees of different age categories. Nurse age bees and food-receiver age bees (6/9- and 12/16-day-old bees respectively) both showed an increase in their GRSs when tested at intervals over the 24 h-stimulation period (K–W: bees of 6–9-days: H = 6.03, p = 0.0491, N = 106; Fig. 6A and 12–16-days: H = 7.53, p = 0.0232, N = 109; Fig. 6B). In both age classes there was a significant increase in GRS after eight hours of stimulation (Dunn comparison: p<0.05, for both age classes; Fig. 6A and 6B), and this level of response was maintained 24 hours after LIO-scented sucrose solution was introduced (Dunn comparison: p<0.05 for 6–9-days; Fig. 6A and p<0.01 for 12–16-days; Fig. 6B). However, foraging bees showed consistent GRSs throughout the test period (K–W: H = 1.105, p = 0.5756, N = 89; Fig. 6C).

### Conditioned response in hive bees

**Figure 7:**
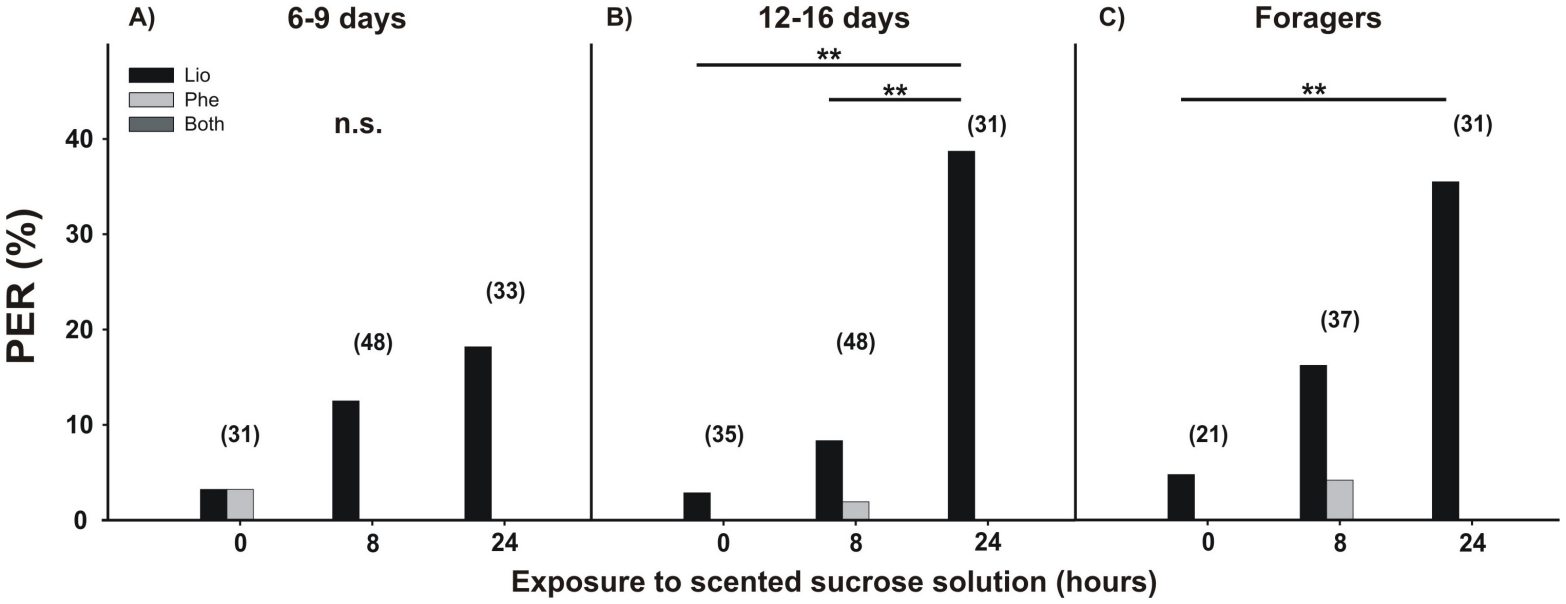
Memory retention of free-flying bees after bees after exposure to scented sucrose solution. The percentage of bees that extended their proboscis on the first presentation of an odor for the solution odor (linalool, LIO, black bars), a novel test odor (phenylacetaldehyde, PHE, gray bars), or both (dark gray bars) were measured in hive bees belonging to different age groups: 6/9 days old (**A**), 12/16 days old (**B**) and foragers (**C**). The asterisks indicate statistical differences in a G-test (** p<0.01, for details see the text). The number of observations is shown in parentheses.

**Figure 8:**
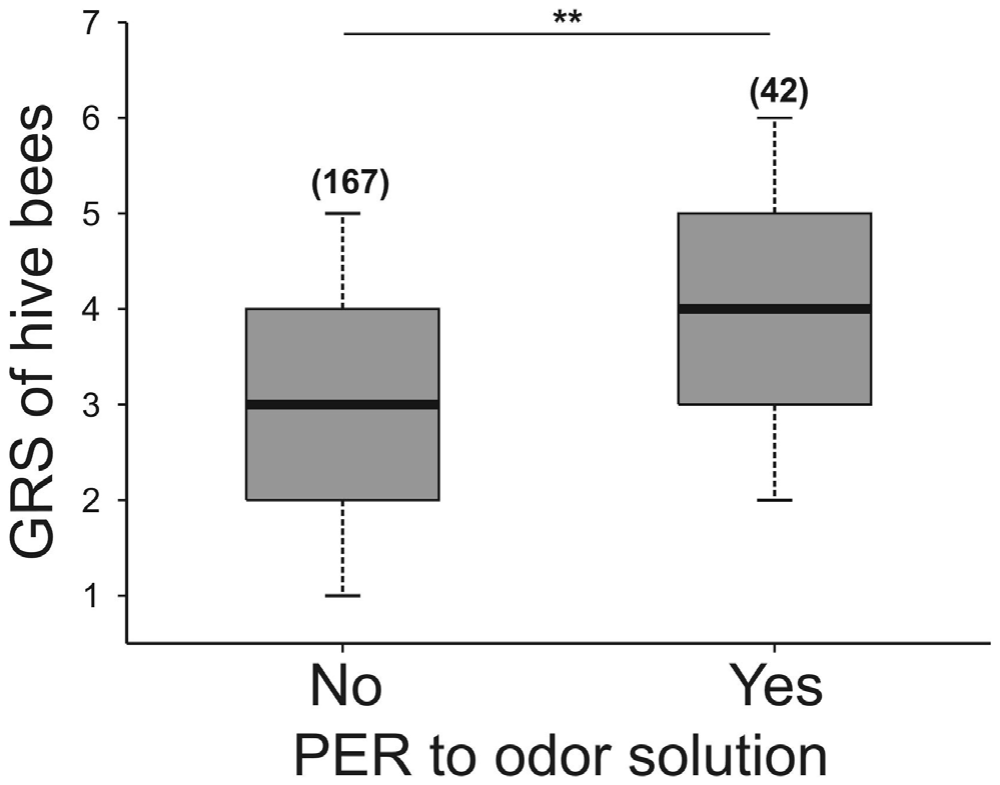
Gustatory responsiveness of free-flying bees after exposure to scented sucrose solution. GRSs of hive bees (bees of 6/9 days old, 12/16 days old and foragers were pooled) were measured while being offered a scented sucrose solution (LIO, 15% w/w). We compared the GRSs of hive bees which extended their proboscis to the first presentation of the sucrose solution odor (LIO) against the other hive bees which did not respond in this way. The asterisks indicate statistical differences in a Kruskal-Wallis test (** p<0.01; for details see the text). The number of observations is shown in parentheses. Boxes indicate the inter-quartile range, horizontal lines within boxes indicate the medians, whiskers include all points within 1.5 times the inter-quartiles, empty circles indicate outliers.

While 6/9-day-old bees showed no significant difference in their PER frequencies between stimulation times (G test: G = 4.12, p = 0.127, N = 112; Fig. 7A), 12/16 days old bees and foragers increased their response levels to LIO over the 24 hour period (G test 12–16: G = 18.03, p = 0.0001, N = 114; Fig. 7B and G test foragers: G = 8.46, p = 0.015, N = 89; Fig. 7C). In both groups there were significant increases in the PER frequency after 24 hours of exposure to LIO-scented sucrose solution in comparison to 0 h (multiple comparison: 12–16 day-bees: p<0.001; Fig. 7B and foragers: p<0.01; Fig. 7C) and 8 h (multiple comparison: 12–16 day-bees: p<0.005; Fig. 7B).

Within the group of bees exposed to scented sucrose solution in their hive, we compared the GRSs of those bees which exhibited a PER to the food odor and those which did not respond (i.e. those which only performed the unconditioned response (UR) to sucrose). Significantly higher GRSs were found for those hive bees that extended their proboscis in response to LIO (Mann-Whitney test, U = 2454, p = 0.002. Bees exhibiting PER, n = 42; bees not exhibiting PER, n = 167; Fig. 8).

### Trophallaxis events of free flying bees to scented and unscented food

**Figure 9:**
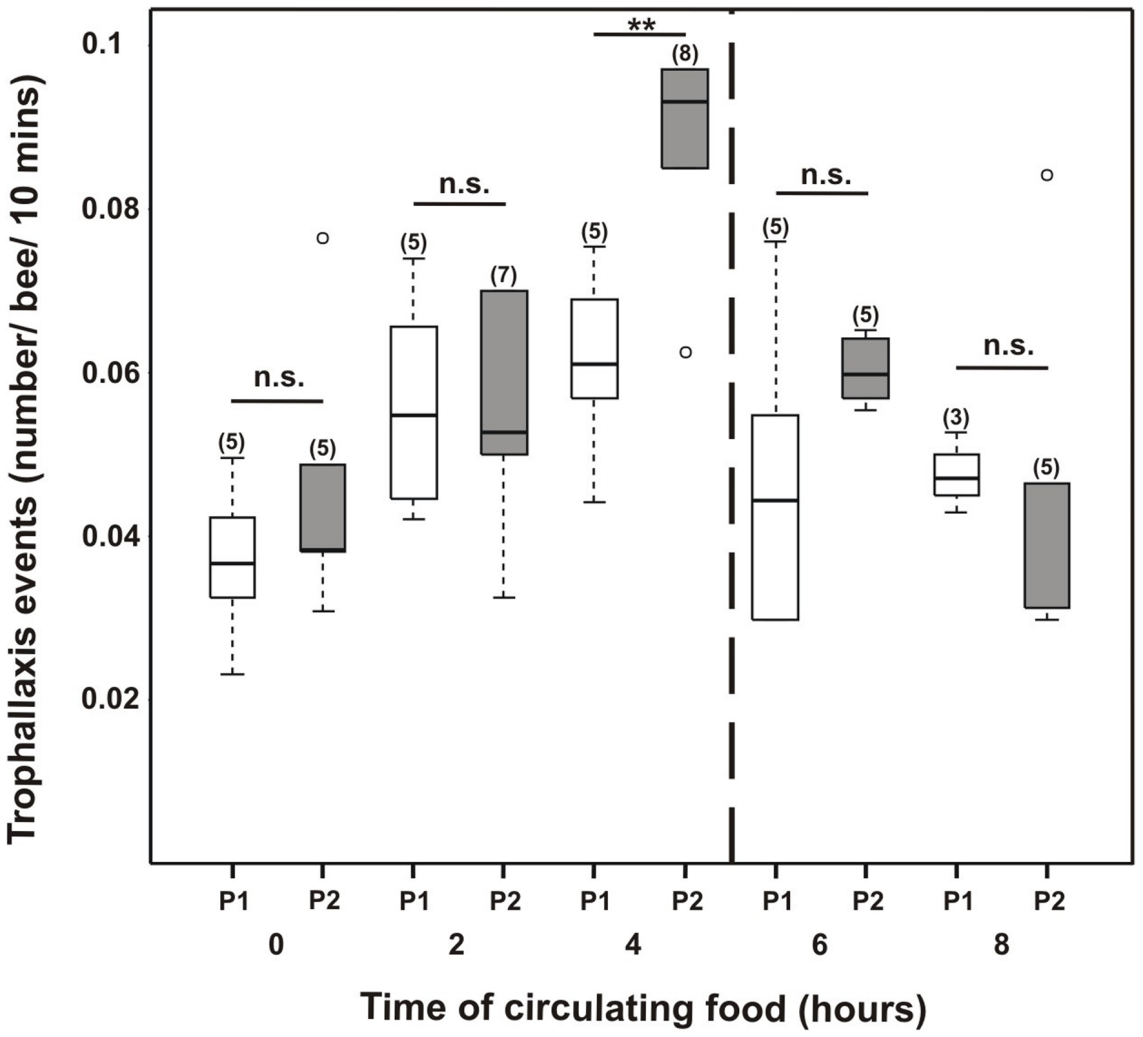
Trophallaxis events in relation to the presence of scent in the circulating nectar. Number of trophallaxis events/bee/10 min of observation from the experimental colony while foragers collected 15% w/w sucrose solution in an artificial feeder placed near the colony for eight (8) hours. White boxes represent the reward program number 1 (P1) in which the colony collected unscented 15% w/w sucrose solution. Gray boxes represent the reward program number 2 (P2) in which bees fed for four (4) hours from a LIO-scented sucrose solution (15% w/w) and afterwards from an unscented solution of the same concentration. The number of observations is shown in parentheses. Boxes indicate the inter-quartile range, horizontal lines within boxes indicate the medians, whiskers include all points within 1.5 times the inter-quartiles, empty circles indicate outliers. The asterisks indicate statistical differences in a Mann- Whitney test (** p<0.01, n.s. not significant; for details see the text).

The number of trophallaxis events occurring in the colony were recorded while foragers were exposed to two reward programs: (i) a constant one in which a 15% w/w unscented sucrose solution was offered during the whole experimental period (control); and (ii) a variable one, in which foragers were fed a 15% w/w LIO-scented sucrose solution during the initial 4 h after which the sucrose solution was replaced by an unscented one. No significant differences were found between the two experimental conditions before foraging for scented food began (Mann-Whitney test: Z = −0.73, p = 0.465, N = 10; Fig. 9), or two hours later (Mann-Whitney test: Z = −0.24, p = 0.807, N = 12; Fig. 9). However, after four hours we observed a significant increase in the number of trophallaxis events performed by bees offered scented sucrose compared to those offered an unscented solution (Mann-Whitney test: Z = −2.64, p = 0.008, N = 13; Fig. 9). Two hours after replacing the scented sucrose solution with an unscented one (6 h after start of the stimulation), the number of trophallaxis events was not different from the control (Mann-Whitney test: Z = −1.57, p = 0.116, N = 10; Fig. 9). Moreover, the number of contacts did not change four hours after the odor was withdrawn (i.e., 8 h after the beginning of the experiment; Mann-Whitney test: Z = 1.04, p = 0.297, N = 8; Fig. 9).

## Discussion

We showed that the gustatory responsiveness of pre-foraging age honeybees was modified after variations in the sucrose content of unscented and scented food, and that there were no changes after exposing bees to a volatile compound in the rearing environment. On the one hand, the observed tuned modulation in responsiveness to sucrose was found in older pre-foraging bees (bees of two weeks of age), which are those that often receive and process the nectar coming into the nest. On the other hand, results showed that younger pre-foraging bees, often located far from the hive entrance and involved in non-foraging related tasks [10], [11], had higher GRSs and appeared to be less able to modulate their responsiveness to sucrose once changes in gustatory and olfactory information occur. Bees were also found to adjust the number of trophallaxis events to the presence of scent in nectar. Taking these observations together with observed differences in the duration of gustatory responsiveness and memory retention for hive bees of different age classes, suggests an enlarged dynamic networking process may occur during nectar exploitation.

### Age-dependent gustatory responsiveness

While one-week-old bees only reduced their GRSs after an increase in sucrose concentration, two-week-old bees modified their GRSs with any change (increase or decrease) in sucrose concentration as well as changes in odors associated with the sucrose solution. Our results indicate that 7-day-old caged bees have significantly higher GRSs than 14-day-old caged bees, a finding which contradicts previous work investigating this trait for bees reared in queen-right colonies [18]. However, recent studies have shown that the rearing environment of honeybees strongly affects odor learning at early ages [33], [37], [38]. These studies show that groups of bees reared in cages and exposed to scented food between five and eight days of age show enhanced memory retention when they reach the foraging age compared with bees exposed to scented food at older pre-foraging ages (bees of 9-/12 days old). These differences are not apparent for bees reared in queen-right colonies. Thus, it could be hypothesized that the higher GRS (i.e. lower sucrose response thresholds) found in one-week-old bees might be one of the factors facilitating the formation of highly stable associative memories at this early adult age [33], [37], [38]. It is worth mentioning that lower sucrose response thresholds correlate with improved learning performance in honeybees conditioned in the PER paradigm [39].

The differences found in responsiveness to sucrose and memory retention between young and old pre-foraging bees could be due to physiological changes in the nervous system and non-neural tissues. Such differences have been noted in circulating hormones and biogenic amines such as the juvenile hormone (JH) and octopamine (OA) throughout the worker's lifespan [40]–[42]. OA and other biogenic amines such as tyramine and dopamine have been identified as behavioral modulators of bees during associative learning [43]–[45] and gustatory responsiveness [19], [46]. The first peak of JH (an OA-related hormone) during worker adulthood is detected a few days after emergence [47]. Interestingly, a related study found high GRSs for 8-day-old bees after topically applying an analog of JH (methoprene), but noted no effect for recently emerged bees treated with the same compound [19].

Gustatory responsiveness of pre-foraging age bees is also associated with foraging choices later in the bee's life [12], [22]. GRSs are modulated by factors such as environmental stimuli [25], nutritional status of the animal [12], and other changes over the honeybee's life related to their behavior [18]. In this sense, it has been reported that there is a tight correlation between the SRTs (GRSs) and the concentration of the incoming nectar in bees within the first week of adult life [12] as well as in older pre-foraging bees which receive the nectar unloaded by returning foragers *via* trophallaxis [16]. Pacheco and Breed [26] have recently reported that middle-aged hive bees (*ca.* 2 weeks old) that undertake non-foraging-related tasks (e.g. fanning, undertaking and guarding) have GRSs similar to those of pollen foragers. These authors hypothesize that such hive bees become pollen foragers. Martinez and Farina [16] showed that nectar receivers (also bees *ca.* 2 weeks old) did not display a sustained low level SRT throughout a four-month foraging season as would be expected for the hive bees that become pollen foragers [21], [26]. This suggests that nectar receivers become nectar foragers, rather than pollen collectors, later in life.

The present study also demonstrates the amount of food-related information that is available in the colony as a result of foraging activity and how each age class responds to these stimuli. The olfactory and gustatory cues circulating within the hive may provide information about the current foraging opportunities to colony members, even to those that have little or no direct contact with foragers ([12] and this study).

### Changes in gustatory responsiveness of free flying bees

A clear reduction in the SRT was found in two-week old bees immediately after an odorant compound was diluted in the sucrose solution offered, in spite of the fact that the presence of odor (diluted in the food) had no impact on the energetic value of the circulating sucrose solution. It has been suggested that olfactory memories formed during food sharing affect the sensitization to sugar, leading to short-lived appetitive arousal [48] and excitation of the animal by lowering the SRT. This response is reinforced by experimental evidence showing that bees exhibiting the PER to the solution odor (CR), also have higher GRSs (lower SRTs) than those that did not respond to the conditioned odor. Note however that this correlation might only be a consequence of different levels of satiation among bees tested [24], [49], [50]. Still, an increase in the GRS occurred only after establishing the olfactory memory in 14-day-old bees and was independent of the quantities of food they ingested (i.e. 14-day-old bees exposed to scented and unscented food consumed similar volumes of sucrose solution in the experimental cages). The increase in GRS after introducing scent only took place when the odorant was diluted in the sucrose solution and not when it was presented as a volatile in the rearing environment. The lack of changes in GRS while bees were exposed to the airborne volatile suggests it is less likely that the observed increases in the GRS could be caused by sensory priming (a biased response to a stimulus on the basis of its previous exposure; [51]). Similarly, since exposure to the volatile compound did not appear to cause a decrease in the GRS, we can reject the possibility of interference of this stimulus with gustatory responsiveness. Honeybees are known to use the olfactory information acquired inside the hive to choose among sources of food outside [33], [52]–[53]. While the presence of scented food circulating in the colony attracts bees to land at feeding sites with this odor [33], [53], a scent exposed as volatiles (airborne) is avoided by bees foraging outside [33]. Ants and bumble bees have also been described to bias their choices according to the foraging context, likely influenced by the odor brought in by a successful forager [54]–[57]. Similar influence of the scent of the food stored in the colony was also found in honeybees [52] and bumble bees [57]–[59].

The fact that the decrease in the GRSs after withdrawing the scented food lasted for at least six hours is difficult to explain within the framework of sensitization to sugar as the only process involved, because sensitization is assumed to occur for a brief period (minutes rather than hours). Also, it is unlikely that residual scented food was still circulating after that time because the solution used was so dilute (15% w/w). This is because a diluted sucrose solution retained in the bee crop passes faster to the gut to be digested than concentrated sucrose solutions [60]. The high CR levels together with the low GRSs (found 24 h after replacing the scented food by an unscented sucrose solution) are additional factors that make it unlikely that these responses are consequences of short lived US properties of the CS [27]. It has been suggested that appetitive learning may produce high levels of reward expectancy (the formation and subsequent activation of memories about specific properties of a given reward; [61]). In this sense, the odor learned during conditioning would not only trigger an appetitive response (such as the extension of the proboscis), but would also promote rewarding properties inducing persistent appetitive arousal.

### Measuring in-hive information propagation through GRS and CR levels

By using a queen-right colony it was possible to observe active foragers achieving high and sustained GRSs, suggesting a high motivational state throughout the stimulation period. The youngest and middle-aged hive bees (12–16 days old) showed increased GRS once the scented sucrose solution began to circulate within the hive. A positive correlation between gustatory responsiveness and odor memory retention was found only in middle-aged bees (12–16 days old bees) showing that for this age group, GRS is highest after scented-food became available. A possible explanation why the younger bees (6–9 day old bees) showed slow increases in their GRSs but not their CRs, is that these workers may be less frequently exposed to multiple odor reward experiences by having fewer (perhaps only one) trophallaxis events. In this case, establishing an appetitive association may be more difficult to achieve [27]. The fact that these bees are located towards the periphery of the network leads them to establish fewer contacts with foragers and unload nectar less often (although 6–9 day old bees have lower response thresholds than 12–16 day old bees). Therefore, the propagation of chemosensory information within the hive, expressed as GRS and CR during the circulation of the scented food, shows clear differences between the various age categories (i.e., high and unchanged behavioral responses for the foragers, an increasing response for middle-aged bees and a lower response for the youngest hive bees).

Although these results clearly show the behavioral plasticity of two-week-old bees in response to changes in chemosensory information, such bees also showed their lowest responsiveness to reward under both experimental conditions. This is particularly relevant since these bees would be the main candidates to start sharing food within the colony after unloading nectar from incoming foragers. Having a high reward threshold implies that the probability of accepting food from successful foragers would be low. With this in mind, it is worth considering how the sharing of the incoming nectar occurs, even when first-receiver bees show a lower acceptance to receive nectar. Hive bees involved in nectar reception are often located in hive areas where communication signals such as dance maneuvers are frequently observed [62]. Therefore, those middle-aged bees might increase their responsiveness to reward by either following dances or staying close to the dance floor. One of the informational components of the honeybee dance is the readiness to respond to information [63], [64]. Thus, a hive bee with low GRS might need more arousal and stimulation *via* this and/or an alternative communication system until it is motivated enough to begin foraging-related tasks; in this particular case, to unload and process the incoming nectar. This makes sense particularly in the light of a study reporting that the honeybee dance attracts not only potential (unemployed) foragers [62], [65], but also food processing bees, which unload the foragers [66].

### Trophallactic changes following colony exposure to scented sucrose solution

Our results suggest it is possible to predict a higher probability of incoming food being accepted (i.e. faster nectar distribution among hive mates), when odor cues are present in the food. Thus, similarly to the exploitation of a profitable resource that would be broadly shared within the colony [4], [5], the increase in trophallaxis events could also be caused by the presence of an odorant cue in the food, even though the shared nectar contained a diluted concentration of sucrose. The increased number of trophallaxis events under the scented food condition could be caused by changes in the gustatory perception of the bees involved, in addition to the presence of an already learned odor inside the colony that could release a CR (i.e. PER to the food odor) in potential food receivers. This mechanism may increase the occurrence of trophallaxis events between incoming foragers and food-receiving bees [17]. The relevance of olfactory cues during the circulation of resources within the hive was further shown by the sudden decrease in trophallaxis events in those assays in which the scent was withdrawn from the food source.

Finally, the differences found in both responsiveness to sucrose and memory retrieval between younger and older pre-foraging hive bees could have implications for the regulation of division of labor during collective foraging. These behavioral differences related to age and current task could imply the presence of individuals within the nest with a high plasticity in their response to changes in resource information, coupled with a low responsiveness to accepting the collected food. These characteristics in nectar receivers would give them not only the role of *hubs* (highly-connected nodes) within the dynamic network, but also of *moderators* which either permit or prevent the passage of the incoming nectar. In contrast, the younger hive bees located at the periphery of the food delivery area (or even further removed) have a higher receptivity to receive food of any quality.

A honeybee colony has the ability to direct its foraging force to the best food sources found in the foraging area [65]. As a consequence, the gustatory and olfactory information acquired by foragers is distributed by individuals of all age groups within the colony [7], [12]. However, the speed and extent with which information is propagated amongst nest mates will depend on the characteristics of the exploited resource in terms of food quality and odorant cues, factors that modulate the sensory-response systems of bees throughout the hive differently. Once a certain type of food is discovered and the collected nectar passes from foragers to the rest of the colony, this modulation could have profound effects on the overall balance between collection and processing capacity of the entire colony in an ever-changing floral market foraging environment.

## References

[1] Newman MEJ (2003). The structure and function of complex networks.. SIAM Review.

[2] Fewell JH (2003). Social insect networks.. Science.

[3] Gordon D (2007). Control without hierarchy.. Nature.

[4] Grüter C, Farina WM (2007). Nectar distribution and its relation to food quality in honeybee (*Apis mellifera*) colonies.. Insectes Soc.

[5] Naug D, Smith B (2007). Experimentally induced change in infectious period affects transmission dynamics in a social group.. Proc R Soc B.

[6] Pírez N, Farina WM (2004). Nectar-receiver behavior in relation to the reward rate experienced by foraging honeybees.. Behav Ecol Sociobiol.

[7] Grüter C, Acosta LE, Farina WM (2006). Propagation of olfactory information within the honeybee hive.. Behav Ecol Sociobiol.

[8] Naug D (2008). Structure of the social network and its influence on transmission dynamics in a honeybee colony.. Behav Ecol Sociobiol.

[9] Rösch GA (1925). Untersuchungen über die Arbeitsteilung im Bienenstaat. 1. Teil: Die Tätigkeiten im normalen Bienenstaate und ihre Beziehungen zum Alter der Arbeitsbienen.. Z vergl Physiol.

[10] Lindauer M (1952). Ein Beitrag zur Frage der Arbeitsteilung im Bienenstaat.. Z vergl Physiol.

[11] Seeley TD (1982). Adaptive significance of the age polyethism schedule in honeybee colonies.. Behav Ecol Sociobiol.

[12] Pankiw T, Nelson M, Page RE, Fondrk MK (2004). The communal crop: modulation of sucrose response thresholds of pre-foraging honey bees with incoming nectar quality.. Behav Ecol Sociobiol.

[13] Farina WM, Grüter C, Diaz PC (2005). Social learning of floral odours inside the honeybee hive.. Proc R Soc B.

[14] Gil M, De Marco RJ (2005). Olfactory learning by means of trophallaxis in *Apis mellifera*.. J Exp Biol.

[15] Farina WM, Grüter C, Acosta L, Mc Cabe S (2007). Honeybees learn floral odors while receiving nectar from foragers within the hive.. Naturwissenschaften.

[16] Martínez A, Farina WM (2008). Honeybees modify gustatory responsiveness after receiving nectar from foragers within the hive.. Behav Ecol Sociobiol.

[17] Goyret J, Farina WM (2005). Non-random nectar unloading interactions between foragers and their receivers in the honeybee hive.. Naturwissenschaften.

[18] Pankiw T, Page RE (1999). The effect of genotype, age, sex, and caste on response thresholds to sucrose and foraging behavior of honey bees (*Apis mellifera* L.).. J Comp Physiol A.

[19] Pankiw T, Page RE (2003). Effect of pheromones, hormones, and handling on sucrose response thresholds of honey bees (*Apis mellifera* L.). J Comp Physiol A.

[20] Roussel E, Carcaud J, Sandoz J-C, Giurfa M (2009). Reappraising social insect behavior through aversive responsiveness and learning.. PLoS ONE.

[21] Page RE, Erber J, Fondrk MK (1998). The effect of genotype on response thresholds to sucrose and foraging behavior of honey bees (*Apis mellifera* L.).. J Comp Physiol A.

[22] Pankiw T, Page RE (2000). Response thresholds to sucrose predict foraging division of labor in honey bees.. Behav Ecol Sociobiol.

[23] Scheiner R, Page RE, Erber J (2001). The effects of genotype, foraging role, and sucrose responsiveness on the tactile learning performance of honey bees (*Apis mellifera* L.).. Neurobiol Learn Mem.

[24] Mujagic S, Erber J (2009). Sucrose acceptance, discrimination and proboscis responses of honey bees (*Apis mellifera* L.) in the field and the laboratory.. J Comp Physiol A.

[25] Pankiw T, Waddington K D, Page R E (2001). Modulation of sucrose response thresholds in honey bees (*Apis mellifera* L.): influence of genotype, feeding and foraging experience.. J Comp Physiol A.

[26] Pacheco J, Breed M D (2008). Sucrose-response thresholds and the expression of behavioural tasks by middle-aged honeybee workers.. Anim Behav.

[27] Menzel R (1999). Memory dynamics in the honeybee.. J Comp Physiol A.

[28] Bitterman ME, Menzel R, Fietz A, Schäfer S (1983). Classical-conditioning of proboscis extension in honeybees (*Apis mellifera*).. J Comp Psychol.

[29] Breed MD, Perry S, Bjostad LB (2004). Testing the blank slate hypothesis: why honey bee colonies accept young bees.. Insectes Soc.

[30] Takeda K (1961). Classical conditioned response in the honey bee.. J Insect Physiol.

[31] Mc Cabe S, Hartfelder K, Santana WC, Farina WM (2007). Odor discrimination in classical conditioning of proboscis extension in two stingless bee species in comparison to Africanized honeybees.. J Comp Physiol A.

[32] Knudsen JT, Tollsten L, Bergstrom LG (1993). Floral scents - A checklist of volatile compounds isolated by headspace techniques.. Phytochemistry.

[33] Arenas A, Farina WM (2008). Age and rearing environment interact in the retention of early olfactory memories in honeybees.. J Comp Physiol A.

[34] Fernández V, Arenas A, Farina WM (2009). Passive volatile exposure within the honeybee hive and its effect on odor discrimination.. J Comp Physiol A.

[35] Sokal RR, Rohlf FJ (2000). *Biometry: the principles and practice of statistics in biological research.*.

[36] Zar JH (1999). *Biostatistical Analysis.* 4th ed.,.

[37] Arenas A, Fernández VM, Farina WM (2009). Associative learning during early adulthood enhances later memory retention in honeybees.. PLoS ONE.

[38] Arenas A, Giurfa M, Farina WM, Sandoz JC (2009). Early olfactory experience modifies neural activity in the antennal lobe of a social insect at the adult stage.. Eur J Neurosci.

[39] Scheiner R, Erber J, Page RE (1999). Tactile learning and the individual evaluation of the reward in honey bees (*Apis mellifera* L.). J Comp Physiol A.

[40] Robinson GE (1992). Regulation of division of labor in insect societies.. Ann Rev Entomol.

[41] Robinson G, Vargo E (1997). Juvenile hormone in adult eusocial Hymenoptera: gonadotropin and behavioral pacemaker.. Arch Insect Biochem.

[42] Bloch G, Sullivan P, Robinson G (2002). Juvenile hormone and circadian locomotor activity in the honey bee *Apis mellifera*.. J Insect Physiol.

[43] Hammer M (1993). An identified neuron mediates the unconditioned stimulus in associative olfactory learning in honeybees.. Nature.

[44] Hammer M, Menzel R (1995). Learning and memory in the honeybee.. J Neurosci.

[45] Vergoz V, Roussel E, Sandoz J-C, Giurfa M (2007). Aversive learning in honeybees revealed by the olfactory conditioning of the sting extension reflex.. PLoS ONE.

[46] Scheiner R, Plückhahns S, Öney B, Blenau W, Erber J (2002). Behavioral pharmacology of octopamine, tyramine and dopamine in honeybees.. Behavioral Brain Research.

[47] Jassim O, Huang ZY, Robinson GE (2000). Juvenile hormone profiles of worker honey bees, *Apis mellifera*, during normal and accelerated behavioural development.. J Insect Physiol.

[48] Hammer M (1997). The neural basis of associative reward learning in honeybees.. Trends Neurosci.

[49] Ben-Shahar Y, Robinson GE (2001). Satiation differentially affects performance in a learning assay by nurse and forager honey bees.. J Comp Physiol A.

[50] Friedrich A, Thomas U, Müller U (2004). Learning at different satiation levels reveals parallel functions for the cAMP-Protein Kinase A cascade in formation of long-term memory.. J Neuroscience.

[51] Schacter DL, Buckner RL (1998). Priming and the brain.. Neuron.

[52] Free JB (1969). Influence of the odour of a honeybee colony's food stores on the behaviour of its foragers.. Nature.

[53] Arenas A, Fernández V, Farina WM (2007). Floral odor learning within the hive affects honeybees' foraging decisions.. Naturwissenschaften.

[54] Roces F (1990). Olfactory conditioning during the recruitment process in a leaf-cutting ant.. Oecologia.

[55] Provecho Y, Josens R (2009). Olfactory memory established during trophallaxis affects food search behaviour in ants.. J Exp Biol.

[56] Dornhaus A, Chittka L (1999). Evolutionary origins of bee dances.. Nature.

[57] Molet M, Chittka L, Raine NE (2009). How floral odours are learned inside the bumblebee (*Bombus terrestris*) nest.. Naturwissenschaften.

[58] Dornhaus A, Chittka L (2001). Food alert in bumblebees (*Bombus terrestris*): possible mechanisms and evolutionary implications.. Behavioral Ecology and Sociobiology.

[59] Dornhaus A, Chittka L (2005). Bumble bees (*Bombus terrestris*) store both food and information in honeypots.. Behavioral Ecology.

[60] Núñez JA, Quesada LA (1971). Repletion of the crop and rectal retention in *Apis mellifera* L.. Physis.

[61] Logan FA (1960). *Incentive*..

[62] Frisch Kvon (1967). *The Dance Language and Orientation of Bees*..

[63] Frisch Kvon (1923). Über die “Sprache” der Bienen. Eine tierpsychologische Untersuchung.. Zool Jahrb, Abt Physiol.

[64] Grüter C, Farina WM (2009). The honeybee waggle dance: can we follow the steps?. Trends in Ecology and Evolution.

[65] Seeley TD (1995). *The Wisdom of The Hive: The Social Physiology of Honey Bee Colonies.*.

[66] Farina WM (2000). The interplay between dancing and trophallactic behavior in the honey bees *Apis mellifera*.. J Comp Physiol A.

